# Clematichinenoside (AR) Attenuates Hypoxia/Reoxygenation-Induced H9c2 Cardiomyocyte Apoptosis via a Mitochondria-Mediated Signaling Pathway

**DOI:** 10.3390/molecules21060683

**Published:** 2016-05-30

**Authors:** Haiyan Ding, Rong Han, Xueshan Chen, Weirong Fang, Meng Liu, Xuemei Wang, Qin Wei, Nandani Darshika Kodithuwakku, Yunman Li

**Affiliations:** 1 State Key Laboratory of Natural Medicines, Department of Physiology, China Pharmaceutical University, Nanjing 210009, China; dinghaiyan79@163.com (H.D.); weirongfang@163.com (W.F.); 2 College of Pharmacy, Xinjiang Medical University, Urumqi 830011, China; ronger051125@163.com (R.H.); chenxueshan2010@sina.com (X.C.); 3 Cancer Hospital Affiliated to Xinjiang Medical University, Urumqi 830011, China; liuxiaomeng198154@163.com; 4 Xinjiang Key Laboratory of Medical Animal Model Research, Clinical Medical Research Institute of the First Affiliated Hospital of Xinjiang Medical University, Urumqi 830011, China; wxuemei1983@sina.com (X.W.); fashion1027@163.com (Q.W.); 5 Institute of Indigenous Medicine, University of Colombo, Rajagiriya 11600, Sri Lanka; darshi_ko@yahoo.com

**Keywords:** clematichinenoside (AR), apoptosis, H9c2 cardiomyocytes, hypoxia/reoxygenation, mitochondria, signaling pathway

## Abstract

Mitochondria-mediated cardiomyocyte apoptosis is involved in myocardial ischemia/reperfusion (MI/R) injury. Clematichinenoside (AR) is a triterpenoid saponin isolated from the roots of *Clematis chinensis* with antioxidant and anti-inflammatory cardioprotection effects against MI/R injury, yet the anti-apoptotic effect and underlying mechanisms of AR in MI/R injury remain unclear. We hypothesize that AR may improve mitochondrial function to inhibit MI/R-induced cardiomyocyte apoptosis. In this study, we replicated an *in vitro* H9c2 cardiomyocyte MI/R model by hypoxia/reoxygenation (H/R) treatment. The viability of H9c2 cardiomyocytes was determined by MTT assay; apoptosis was evaluated by flow cytometry and TUNEL experiments; mitochondrial permeability transition pore (mPTP) opening was analyzed by a calcein-cobalt quenching method; and mitochondrial membrane potential (ΔΨm) was detected by JC-1. Moreover, we used western blots to determine the mitochondrial cytochrome c translocation to cytosolic and the expression of caspase-3, Bcl-2, and Bax proteins. These results showed that the application of AR decreased the ratio of apoptosis and the extent of mPTP opening, but increased ΔΨm. AR also inhibited H/R-induced release of mitochondrial cytochrome c and decreased the expression of the caspase-3, Bax proteins. Conversely, it remarkably increased the expression of Bcl-2 protein. Taken together, these results revealed that AR protects H9c2 cardiomyocytes against H/R-induced apoptosis through mitochondrial-mediated apoptotic signaling pathway.

## 1. Introduction

Ischemia/reperfusion is associated with thrombolysis, angioplasty and coronary bypass surgery which relieve myocardial ischemia, but simultaneously cause further damage to myocardial tissue, which is known as myocardial ischemia/reperfusion (MI/R) injury [1]. MI/R injury is one of the death threats in various cardiovascular diseases [2]. During MI/R injury, cardiomyocytes undergo apoptosis [3,4], which results in myocardial contractile dysfunction, compensatory hypertrophy and reparative fibrosis, all of which further increase myocardial injury, and ultimately leads to cardiac dysfunction [5]. Apoptosis is considered one of the key pathological processes in MI/R injury and the amount of apoptosis determines the severity of MI/R injury [6]. Therefore, deciphering the mechanisms by which the cardiomyocytes apoptosis in MI/R injury has an important impact on the development of effective therapeutics [7,8].

Apoptosis is a process of programmed cell death and can be induced by several signaling pathways, in which the mitochondrial pathway plays an important role and mediates the apoptotic modulation in the whole process [6]. Mitochondrial-mediated apoptotic pathway can be triggered by several factors. One is the opening of mitochondrial permeability transition pore (mPTP), a large, nonselective conductance pore located in the inner mitochondrial membrane. mPTP exerts a crucial effect in triggering apoptosis of cardiomyocytes exposed MI/R injury [9]. Once the mPTP is open, mitochondrial membrane potential (ΔΨm) is depolarized, leading to pro-apoptotic factors leakage cascade reactions and mitochondrial dysfunction. Cytochrome c, one of the pro-apoptotic factors, has been regarded a decisive step to initiate apoptosis in MI/R injury [10]. The leakage of cytochrome c promotes caspase activation, in which caspase-3 is the key caspase responsible for promoting cell death [11]. Additionally, Bcl-2 family proteins affect the permeability of the mitochondrial membrane in MI/R-induced apoptosis [12]. Therefore, ameliorating mitochondrial function is an effective therapeutic strategy to reduce apoptosis and protect the heart from MI/R injury.

**Figure 1:**
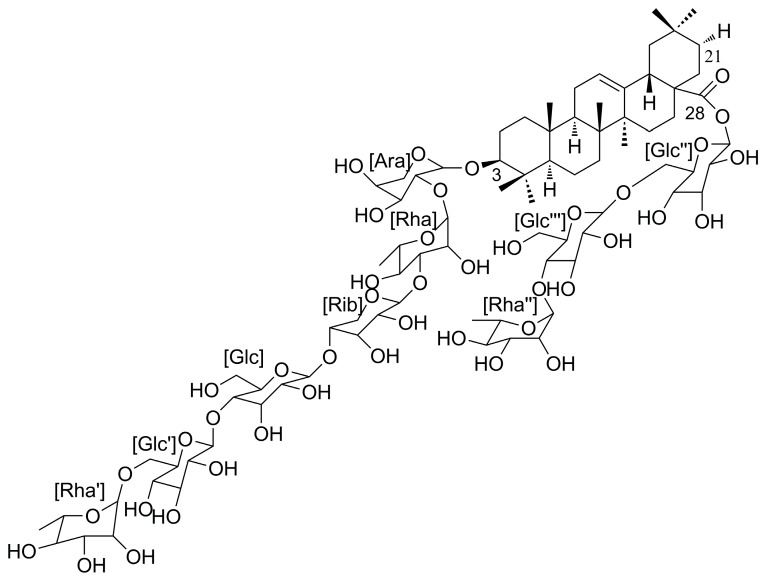
Chemical structure of clematichinenoside (AR).

Clematichinenoside (AR, Figure 1, 3-*O*-[α-l-rhamnopyranosyl-(1→6)-β-d-glucopyranosyl-(1→4)-β-d-glucopyranosyl-(1→4)-β-d-ribopyranosyl-(1→3)-α-l-rhamnopyranosyl-(1→2)-α-l-arabinopyranosyl]- oleanolic acid 28-*O*-[α-l-rhamnopyranosyl-(1→4)-β-d-glucopyranosyl-(1→6)-β-d-glucopyranosyl] ester), is a major bioactive component isolated from the roots of *Clematis chinensis*, whose isolation procedure has been discussed by Liu *et al.* [13]. Clematichinenoside has anti-arthritic and anti-inflammatory effects, and can attenuate myocardial infarction following MI/R injury [14,15,16]. However, whether AR has anti-apoptotic effects on MI/R-induced cardiomyocyte apoptosis remains unexplored. We hypothesized that AR may improve the mitochondria function to inhibit MI/R-induced cardiomyocyte apoptosis. Therefore, the aims of the present study were to investigate whether AR can indeed inhibit MI/R-induced cardiomyocytes apoptosis and to decipher the mechanisms underlying the mitochondrial-mediated apoptotic signaling pathway.

## 2. Results

### 2.1. AR Pretreatment Increases Cell Viability

**Figure 2:**
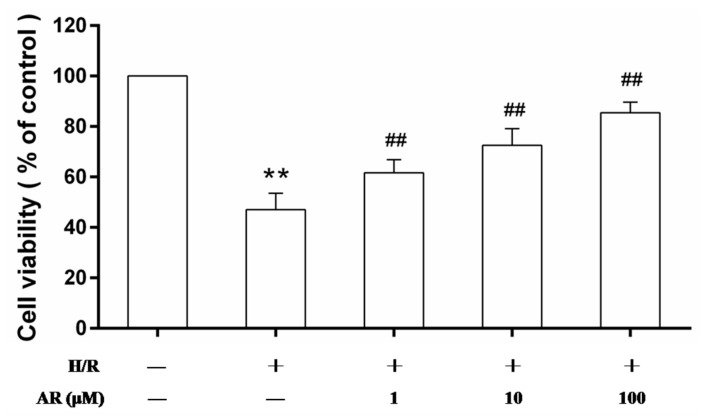
Effects of AR on H9c2 cardiomyocytes viability. After treatment with AR (1, 10, 100 μM) for 24 h, H9c2 cardiomyocytes were treated with H/R injury. Cells viability was analyzed by [3-(4,5-dimethylthiazol-2-yl)-2,5-diphenyltetrazolium bromide] (MTT) assay, and the value of the control group was considered 100%. Values presented are mean ± S.D (*n* = 8). **: *p* < 0.01 *vs.* control group; ^##^: *p* < 0.01 *vs.* H/R group.

Viable cells were reduced with H/R treatment and accounted for 47.1% of the control group. Compared with H/R group, cell viability was increased significantly in AR (1, 10, 100 μM) pretreatment groups from 47.1% to 61.7%, 72.6% and 85.4%, respectively (Figure 2). The results suggested that AR owned protective effect in H9c2 cardiomyocytes against H/R injury in a concentration-dependent manner.

### 2.2. AR Pretreatment Inhibits H9c2 Cardiomyocytes Apoptosis against H/R Injury

**Figure 3:**
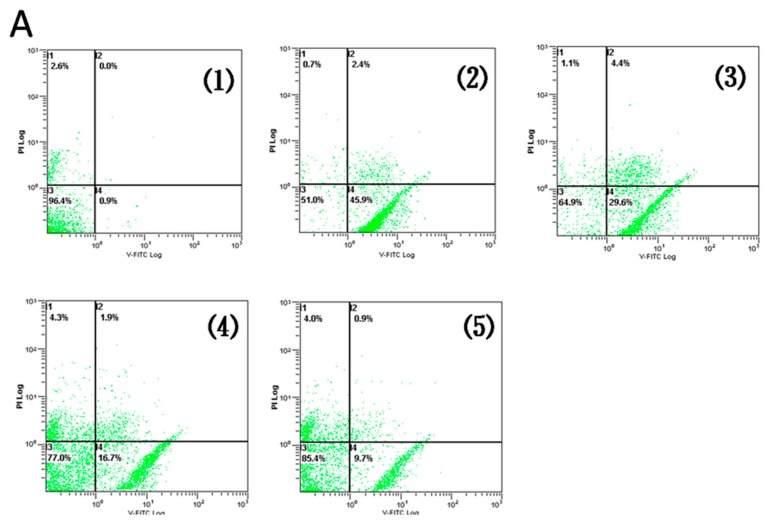

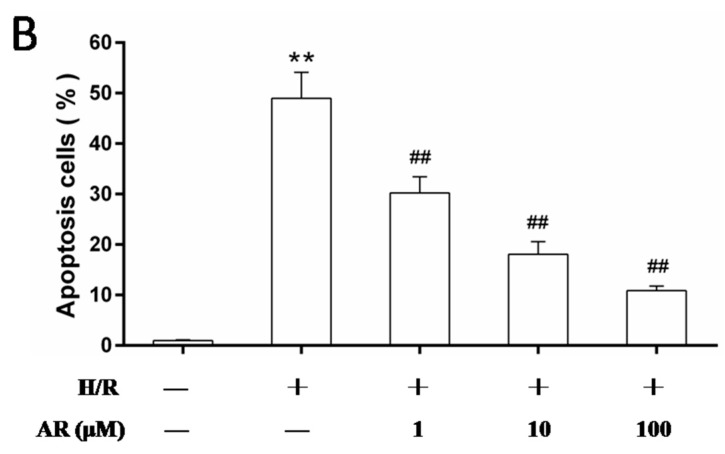
Effects of AR on H/R-induced H9c2 cardiomyocytes apoptosis. Flow cytometry analysis was carried out to determine apoptotic ratio in experimental groups. (**A**): (1) Control group; (2) H/R group; (3) AR low-dose (1 μM) group; (4) AR intermediate-dose (10 μM) group; (5) AR high-dose (100 μM) group; (**B**) The histogram shows the relative proportion of apoptosis cells in different experimental groups. Values presented are mean ± S.D (*n* = 5). **: *p* < 0.01 *vs.* control group; ^##^: *p* < 0.01 *vs.* H/R group.

In order to investigate whether AR protects H9c2 cardiomyocytes against H/R-induced apoptosis, cells were detected by Annexin V-FITC/PI double staining (Figure 3).

Only Annexin V-FITC positive but PI negative cells were considered as early apoptotic cells, which were defined as the quantity. Flow cytometry analysis results demonstrated that there was significant elevation of apoptosis in H/R group compared to control (49.0% *vs.* 0.98%, *p* < 0.01). While apoptosis reduced remarkably in pretreatment with AR (1, 10, 100 μM) compared with H/R group (*p* < 0.01, Figure 3B). The results showed pretreatment of AR inhibited H/R-induced apoptosis in H9c2 cardiomyocytes.

### 2.3. AR Pretreatment Protects H9c2 Cardiomyocytes against H/R Injury

**Figure 4:**
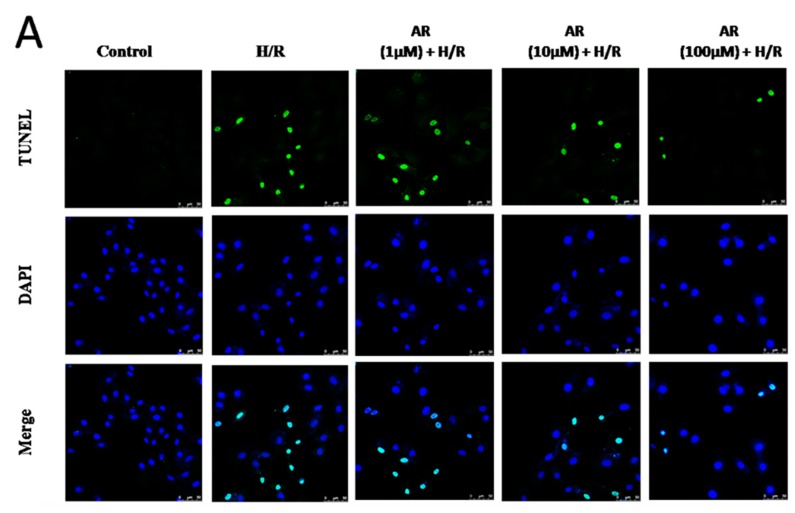

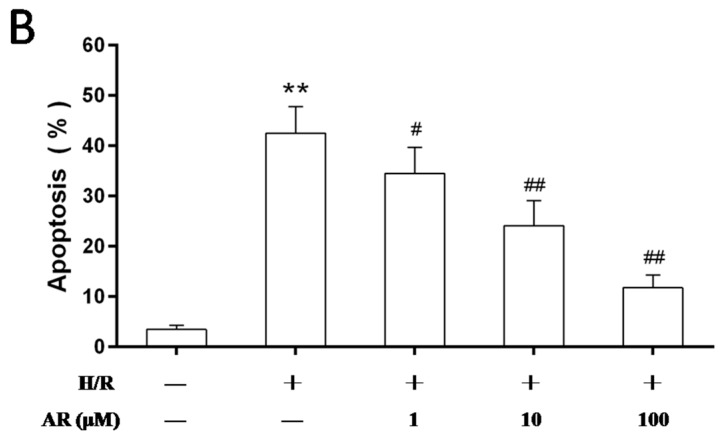
Protection effects of AR against H/R-induced H9c2 cardiomyocytes apoptosis. Pretreatment with AR (1, 10, 100 μM) for 24 h prior to H/R, morphology and apoptosis of H9c2 cardiomyocytes were assessed by DAPI and TUNEL staining. (**A**) Representative images of TUNEL-positive cells (green, first row) and DAPI counterstaining (blue, middle row). Scale bar: 50 μm; (**B**) The histogram shows the relative proportion of TUNEL-positive cells in experimental groups. Values are mean ± S.D (*n* = 5). **: *p* < 0.01 *vs.* control group; ^#^: *p* < 0.05 *vs.* H/R group; ^##^: *p* < 0.01 *vs.* H/R group.

To further determine the protective role of AR on H/R-induced apoptosis in H9c2 cardiomyocytes, we observed cell morphology by nuclear morphology by DAPI and terminal deoxynucleoitidyl transferase-mediated biotinylated UTP nick end labeling (TUNEL) staining. As shown in Figure 4A, DAPI staining showed that regular contour and round or elliptical nuclei existed in normal cells.

In contrast, cells with H/R-treated appeared with smaller nuclei and shrunk chromatin. Pre-administration of AR significantly improved cell morphology and reduced the number of H/R-induced apoptosis. The apoptosis ratio was higher in H/R group compared to control group (42.5% *vs.* 3.5%, *p* < 0.01, Figure 4B). Compared with H/R group, AR (1, 10, 100 μM) decreased H/R-induced apoptosis from 42.5% to 34.5%, 24.1% and 11.8% (*p* < 0.05, *p* < 0.01 and *p* < 0.01 respectively). The results illustrated that AR possessed the effect not only protected cell morphology but also reduced H/R-induced apoptosis.

### 2.4. AR Pretreatment Inhibits mPTP Opening Induced by H/R Injury

**Figure 5:**
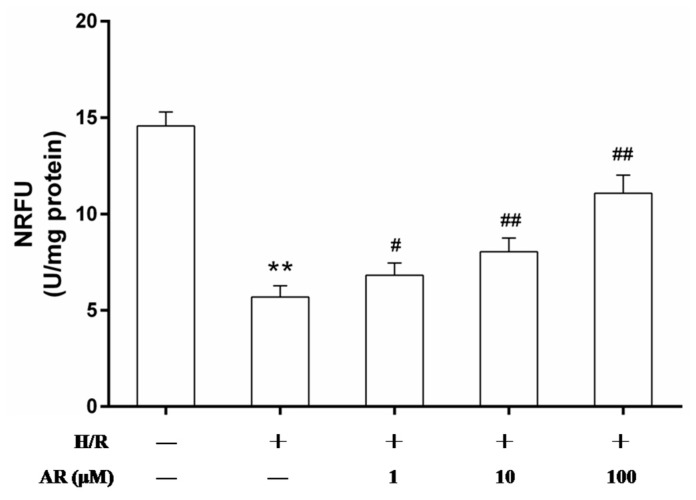
AR pretreatment inhibited the mPTP opening in H/R-injured H9c2 cardiomyocytes. The mPTP opening was detected by the calcein–cobalt method. All experimental groups were measured the relative normalized fluorescent units (NRFU) of calcein. Values are mean ± S.D (*n* = 5). **: *p* < 0.01 *vs.* control group; ^#^: *p* < 0.05 *vs.* H/R group; ^##^: *p* < 0.01 *vs.* H/R group.

To investigate whether AR inhibits mitochondrial permeability transition pore (mPTP) opening, we detected the mPTP opening mode with the calcein-cobalt method. Compared with the control, we observed that fluorescence significantly decreased in H/R cells (*p* < 0.01). However, pretreatment of AR (1, 10, 100 μM) demonstrated a significant higher level compared with H/R group (*p* < 0.05, *p* < 0.01 and *p* < 0.01 respectively, Figure 5). These results demonstrated that AR owned the ability to reduce the extent of mPTP opening of H/R-injured cells in response to the protection effect of mitochondria.

### 2.5. AR Pretreatment Maintains the Mitochondrial Membrane Potential (ΔΨm) in H/R-Treated Cells

**Figure 6:**
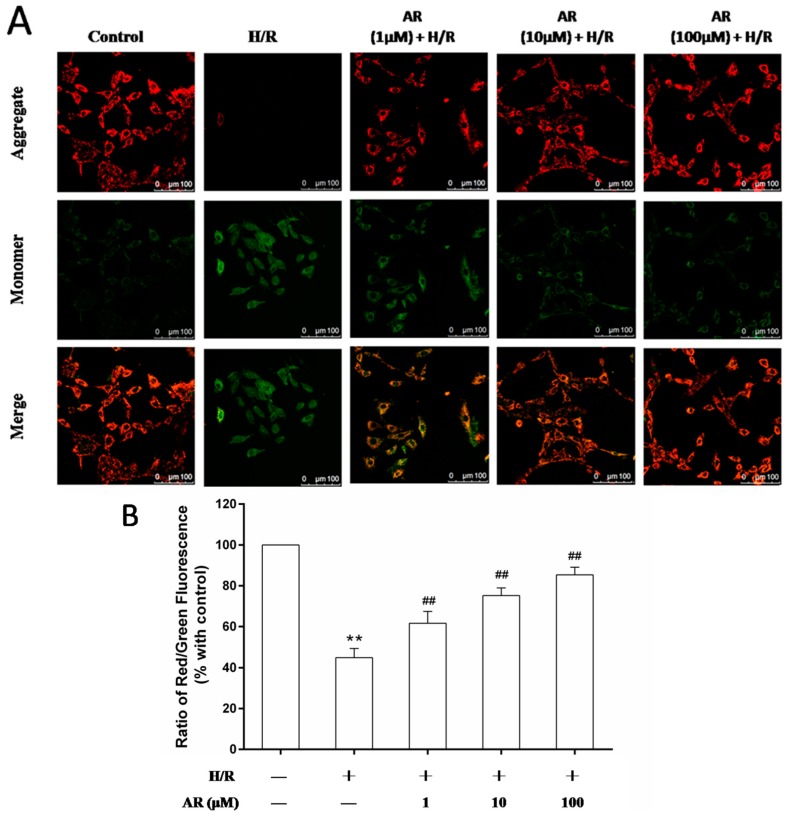
AR pretreatment maintained the ΔΨm in H/R-injured cells. ΔΨm of the different groups was estimated with JC-1 probe. For red fluorescent excitation is 585 nm and emission is 590 nm; for green fluorescent excitation is 514 nm and emission is 530 nm. (**A**) JC-1 aggregates red fluorescence in mitochondria of normal cells. JC-1 accrues as green fluorescence monomer in the cytosol of apoptotic cells, which indicates ΔΨm collapsed. Merged images showed the co-localization of JC-1 aggregates and monomers. Scale bar: 100 μm; (**B**) The ΔΨm of cells in different groups was calculated as the fluorescent ratio of red to green. Values are mean ± S.D (*n* = 5). **: *p* < 0.01 *vs.* control group; ^##^: *p* < 0.01 *vs.* H/R group.

To further assess the role of AR on the mitochondrial function, ΔΨm was measured by using the 5,5′,6,6′-tetrachloro-1,1′,3,3′-tetrathylbenzimidazol carbocyanine iodide (JC-1) probe. ΔΨm plays a critical function in cell life. Maintaining ΔΨm is essential to preserve mitochondrial function. The collapse of the ΔΨm is an important sign of apoptosis occurrence. As shown in Figure 6A, lower level of red fluorescence and higher level of green fluorescence were observed in H/R-treated cells, but pretreatment of AR (1, 10, 100 μM) reversed the fluorescence changes (*p* < 0.01). The ΔΨm was calculated as the fluorescent ratio of red to green. The lower ratio illustrated the level of mitochondrial depolarization (Figure 6B). These results revealed that pretreatment of AR prevented the mitochondrial depolarization and maintained the mitochondrial intact. Moreover, it suggested that the protective role of AR against H/R-injured apoptosis associated with mitochondria function.

### 2.6. AR Pretreatment Prevents Cytochrome c Release

**Figure 7:**
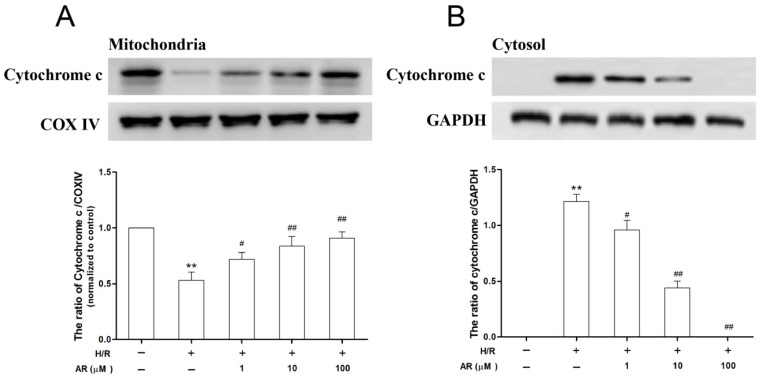
Effects of AR on preventing cytochrome c release in H9c2 cardiomyocytes exposed to H/R. (**A**) The release of cytochrome c in mitochondria; (**B**) Cytosolic translocation of mitochondrial cytochrome c in different experimental groups. Values presented are mean ± S.D (*n* = 3). **: *p* < 0.01 *vs.* control group; ^#^: *p* < 0.05 *vs.* H/R group; ^##^: *p* < 0.01 *vs.* H/R group.

Leakage of cytochrome c from mitochondria to cytosol is an important sign in cell apoptotic pathway after mPTP opening and ΔΨm collapse. In the present study, cytochrome c released from mitochondria was determined by western blot. Compared with the control, mitochondrial membranes damage was severe in H/R group, leading to the leakage of cytochrome c from mitochondria. There was significant decrease of cytochrome c in the mitochondria in H/R-treated cells (*p* < 0.01, Figure 7A). However, cytochrome c in the mitochondria was increased significantly with pretreatment of AR (1, 10, 100 μM) compared with H/R group (*p* < 0.05, *p* < 0.01 and *p* < 0.01 respectively). Intriguingly, cytochrome c was observed only in cytosol of H/R, AR low-dose groups and intermediate-dose groups. Furthermore, there was significant difference between H/R and AR low-dose, intermediate-dose groups (*p* < 0.05, *p* < 0.01, Figure 7B). These results showed that cytochrome c release was efficiently inhibited by AR pretreatment in H/R-injured H9c2 cardiomyocytes, which revealed that AR may protect cardiomyocytes apoptosis potentially by regulating the mitochondrial apoptotic pathway.

### 2.7. AR Pretreatment Suppresses Caspase-3 Activity and Increases the Ratio of Bcl-2 to Bax Exposed to H/R Injury

**Figure 8:**
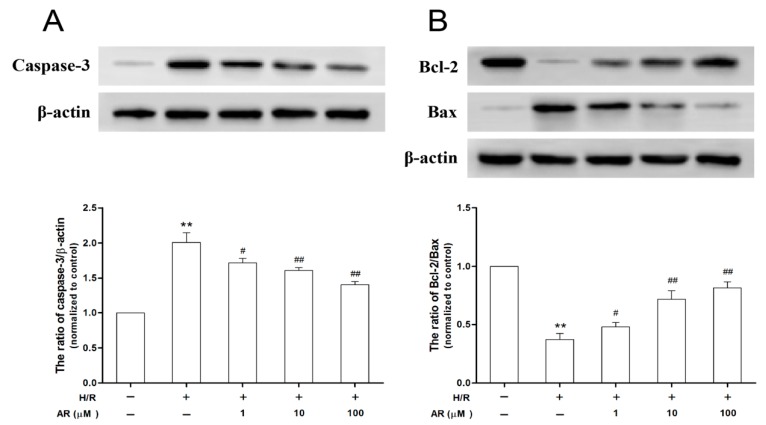
AR pretreatment suppressed the activity of casepase-3 and enhanced the ratio of Bcl-2 to Bax in H9c2 cardiomyocytes subjected to H/R. (**A**) Casepase-3 expression of different groups; (**B**) The ratio of Bcl-2 to Bax in various experimental treatment groups. Values presented are mean ± S.D (*n* = 3). **: *p* < 0.01 *vs.* control group; ^#^: *p* < 0.05 *vs.* H/R group; ^##^: *p* < 0.01 *vs.* H/R group.

Caspase-3 plays a determinant role in apoptotic progress. Compared with the control, expression of caspases-3 was significantly promoted in H/R group (*p* < 0.01), while pretreatment of AR (1, 10, 100 μM) reversed these increases significantly (*p* < 0.05, *p* < 0.01 and *p* < 0.01 respectively, Figure 8A). These results demonstrated that the beneficial effects of AR were accompanied with the suppression of the caspases-3 activity in H/R-injured H9c2 cardiomyocytes.

Bcl-2 and Bax proteins, the Bcl-2 family proteins, serve important roles in triggering the mitochondrial death cascade. In the present study, the expression of Bcl-2 and Bax proteins were measured by western blot. As shown in Figure 8B, in H/R group Bcl-2 expression decreased and Bax expression increased significantly compared with control group. However, pretreatment of AR (1, 10, 100 μM) up-regulated Bcl-2 expression, suppressed Bax expression and enhanced the Bcl-2/Bax ratio significantly compared with the H/R group (*p* < 0.05, *p* < 0.01 and *p* < 0.01 respectively). These results suggested that AR protected against H/R-induced apoptosis in H9c2 cardiomyocytes by modulating the balance of Bcl-2 and Bax proteins.

## 3. Discussion

It is well known that mitochondria not only control the energy metabolism of the body, but also regulate cell apoptosis or necrosis [17]. Increasing evidence suggests that MI/R-induced cell apoptosis is closely associated with mitochondria dysfunction [18,19]. Therefore, ameliorating the mitochondrial function is an effective method to attenuate the H/R-induced injury in cardiomyocytes. Based on these notions, we hypothesized that the cardioprotection of AR may connect with regulating mitochondrial function. Zhang *et al.* have reported that AR attenuates myocardial infarction in H/R injury by its antioxidant effects and by restoring the balance of nitric oxide synthase [16]. Our previous study found that AR has anti-arthritic effects through PI3K/Akt signaling pathway and attenuating TNF-α associated collagen-induced arthritis [14]. Recently, we found that AR alleviates cerebral inflammatory injury through A20-NF-κB signal pathway [20]. The present study revealed that the application of AR increases the cell viability and reduces H/R-induced cardiomyocyte apoptosis through improving mitochondria function, demonstrated by inhibiting mPTP opening, maintaining mitochondrial permeability transition pore (ΔΨm), preventing cytochrome c leakage from mitochondria to cytoplasm, suppressing caspase-3 activity, and increasing the ratio of Bcl-2 to Bax exposed H/R injury. All of which are closely associated to mitochondria-dependent signaling pathways. These results further verified our previous hypothesis. To the best of our knowledge, the present study has elucidated for the first time that the mitochondria-mediated signaling pathway is one of the mechanisms underlying AR protects the H/R-injury induced cardiomyocyte apoptosis.

mPTP exerts a crucial role in triggering the apoptosis of cardiomyocytes exposed H/R injury [9]. Once mPTP is open, mitochondrial membrane becomes sensitive, resulting in dissipation of membrane potential, that in turn affecting the role of mitochondria [21]. ΔΨm is another important factor regulating mitochondrial function, and acts as an indicator of the status of mPTP [22]. Intriguingly, we showed that AR significantly alleviates the H/R injury-induced mPTP opening and ΔΨm depolarization in a dose-dependent manner. This result is consistent with the finding that lycopene exerts its anti-apoptotic effect through suppressing mPTP opening to inhibit mitochondrial dysfunction [23].This suggests that cardioprotection of AR during H/R injury is tightly associated with normal function of mitochondria.

During H/R injury, along with mPTP opening and ΔΨm depolarization mitochondrial dysfunction is inevitable, and then pro-apoptotic factors, such as cytochrome c, are released, which has been considered as a decisive procedure to activate the mitochondrial apoptotic pathway [10]. Furthermore, the leakage cytochrome c promotes caspase activation, which executes and controls apoptotic signaling [24]. Among various caspases, caspase-3 is the key caspase and responsible for promoting cell death [11]. Consistent with these results, we indicated that increases of the leakage cytochrome c and caspase-3 activity were detected in H/R treatment. However, this study showed that the leakage of cytochrome c from mitochondria to cytoplasm and the activity of caspase-3 were significantly attenuated by a pretreatment with AR dose-dependently. All these findings demonstrated that AR closely associates with the functional regulation of mitochondrial, through which preventing H/R injury-induced cardiomyocyte apoptosis.

Bcl-2 family is a kind of apoptosis related genes, its expression and regulation are the key factors influencing apoptosis [25]. Bcl-2/Bax ratio is another important parameter in mitochondria signaling pathway. Alteration of the Bcl-2/Bax ratio influences apoptotic balance [26,27,28]. Bcl-2 family proteins play an important role in apoptosis by regulating the permeabilization of the mitochondrial membrane [12]. In our study, we had another novel discovery that AR can significantly increase Bcl-2 and decrease Bax protein expression, therefore enhancing the Bcl-2/Bax ratio in a concentration-dependent manner compared with H/R group. Bcl-2 regulates the opening and closing of mitochondrial permeability transition pore (mPTP) to exert an anti-apoptosis role, but Bax plays a pro-apoptosis function in the family. The evidence could indicate the up-regulation the ratio of Bcl-2/Bax in AR pretreatment group, which consists with our finding that AR protects cardiomyocytes against H/R injury through mitochondrial pathway. Cook et al have reported that Bcl-2 regulates mPTP opening in opposition to Bax, prevents cytochrome c leakage, suppresses caspase activity and decreases cell apoptosis [29,30], which are consistent with our results that the anti-apoptosis protein Bcl-2 is related to mitochondria. Therefore, we validate that up-regulating Bcl-2 and down-regulating Bax by AR are connected with mitochondria-dependent cardioprotection in H/R-injured cardiomyocytes. However, the underlying mechanism is not fully clear and needs further study.

## 4. Experimental Section

### 4.1. Materials

Clematichinenoside (AR, 95.3% purity, required concentration prepared with PBS before use) was prepared by the School of Traditional Chinese Pharmacy, China Pharmaceutical University (Nanjing, China). Foetal Bovine Serum (FBS) and Dulbecco’s modified Eagle’s medium (DMEM) were products of Hyclone Co. (Logan, UT, USA). Trypsin, penicillin and streptomycin were products of Grand Island Biological Company (Gibco, Grand Island, NY, USA). We purchased RIPA Buffer from Thermo Scientific Co. (Waltham, MA, USA); anti-Bcl-2 and anti-Bax from Cell Signaling Technology (Danvers, MA, USA); anti-cytochrome c and anti-caspase-3 from USCN Business Co. (Wuhan, China); anti-β-actin and the horseradish peroxidase-labeled IgG secondary antibodies from Boster Biological Technology (Wuhan, China). All other reagents were analytical grade and commercially available.

### 4.2. Cell Culture

The H9c2 cell line was provided by Boster Biological Technology and cultured with regular medium (DMEM with 15% FBS and 100 U/mL of penicillin and streptomycin) in humidified incubator of 5% CO_2_ at 37 °C. Two days later, H9c2 cardiomyocytes grew into contractile layers, and the experiments of drug treatment were performed to induce the model of H9c2 cardiomyocytes hypoxia/reoxygenation (H/R) damage [16,31]. H9c2 cardiomyocytes were randomly and equally assigned into the five groups with different treatments. (1) Control group: No defined specific treatment; (2) H/R group: 5mM Na_2_S_2_O_4_ in the regular medium for 2 h (hypoxia) and then without Na_2_S_2_O_4_ (reoxygenation) for 2 h; (3) Low-dose (1 μM) AR plus H/R group; (4) Intermediate-dose (10 μM) AR plus H/R group; (5) High-dose (100 μM) AR plus H/R group. The treatment duration of various doses of AR was 24 h, and then the treated cardiomyocytes were washed twice with PBS before H/R application.

### 4.3. Detect Cell Viability by MTT Assay

H9c2 cardiomyocytes were cultured and pretreated with the final concentrations of 1, 10, 100 μM AR for 24 h prior to H/R treatment. After that, MTT (0.5%, 20 μL) was added to the medium for 4 h at 37 °C in the dark. The supernatant was removed, and dimethyl sulfoxide (DMSO, 100 μL /well) was used to dissolve the precipitate. The absorbance was detected at 490 nm with a Multiskan Spectrum instrument (MK3, Thermo Scientific).

### 4.4. Determination of Apoptosis by Flow Cytometry

H9c2 cardiomyocytes were pretreated with concentrations of 1, 10, 100 μM AR for 24 h before H/R treatment, respectively. After centrifugation, cells (1 × 10^6^) were resuspended in labeling solution for 15 min at room temperature in the dark. Cell fluorescence and apoptosis were analyzed by flow cytometry (Beckman ALTRA, Brea, CA, USA). Living cells, apoptotic cells in early or late phase of apoptosis were identified in quadrant dot plot. The Annexin V-FITC and PI (Roche, Los Angeles, CA, USA) double negative stained cells were identified as living cells. Only Annexin V–FITC positive cells were considered as early apoptotic cells and double positive staining cells were the late apoptosis cells. Apoptosis ratio was recognized as the percentage of Annexin V–FITC-positive cells.

### 4.5. Determination of Apoptosis by TUNEL Assay

To further observe the morphological difference between normal cells and apoptotic cells, apoptosis was also detected by TUNEL assay using an *in situ* cell death detection kit (Roche) according to the manufacturer’s protocol. H9c2 cardiomyocytes were finally counterstained with DAPI (Sigma, St. Louis, MO, USA) for 5 min at room temperature, and examined using a TCS SP8 confocal laser scanning microscope (Leica, Solms, Germany). Four non-overlapping fields of vision were observed in each confocal dish. Apoptotic percentage was the ratio of TUNEL-positive nuclei to the total cell nuclei counterstaining by DAPI.

### 4.6. The Opening of Mitochondrial Permeability Transition Pore (mPTP) Detection

Transient mPTP opening was directly detected by using a mPTP assay kit (Genmed Scientifics Inc., Arlington, MA, USA) as described previously [32]. Briefly, in bottom 24 hole plate, H9c2 cardiomyocytes were rinsed with Reagent A, then treated with Reagents B and C (1:100; 500 μL/well) at 37 °C for 20 min in the dark, after that rinsed twice in Reagent A. Fluorescent intensity was detected using a Varioskan Flash spectrofluorimeter (Thermo Scientific) at λ_ex_ 488 nm, λ_em_ 505 nm. Subsequently, cells were lysed and protein concentration was detected by Bradford Protein (Beyotime, Shanghai, China) assay. The fluorescence was normalized to total protein content. Results were represented as normalized relative fluorescence units (NRFU; U/mg protein).

### 4.7. Measurement of Mitochondrial Membrane Potential (ΔΨm)

ΔΨm of H9c2 cardiomyocytes was measured by cationic probe JC-1 (Beyotime). JC-1 aggregates red fluorescence in mitochondria of normal cells. JC-1 accrues as green fluorescence monomer in the cytosol of apoptotic cells. ΔΨm was increased linearly corresponds to the formation of JC-1 aggregates and their fluorescence [33]. Briefly, H9c2 cardiomyocytes were washed twice with PBS, and then loaded with JC-1 at 37 °C for 20 min. After rinse twice in staining buffer, images were obtained using a Leica confocal laser scanning microscope (TCS SP8). Green and red fluorescent intensity was measured by Varioskan Flash (Thermo Scientific). The ΔΨm was calculated as the fluorescent ratio of red *vs.* green, which indicated the ΔΨm loss.

### 4.8. Analysis of Cytochrome c Leakage from Mitochondrial

Cytochrome c level was determined in cytosol and isolated mitochondria. Isolated mitochondrial was prepared in accordance with instructions of the Mitochondria/Cytosol Isolation Kit (Sangon Biotech, Shanghai, China) as mentioned previously [34]. In brief, cells were collected and homogenized about 50 times with ice-cold Mito-Cyto Buffer in Dounce homogenizer. Following centrifugation, the supernatant was gathered and then centrifuged, which was cytosol protein. The precipitation was resuspended in Mito-Cyto Buffer to obtain the protein of mitochondrial. After Bradford protein assay, cytochrome c was analyzed by western blot (Figures S1–S4 of Supplementary Materials).

### 4.9. Western Blot Analysis for Caspase-3, Bcl-2 and Bax

H9c2 cardiomyocytes total proteins were prepared as depicted previously [35,36]. Cultured cells were rinsed twice in cold PBS and immersed in RIPA buffer on ice for 5 min, and then lysates were gathered and centrifuged. Following protein concentration quantitation, denatured protein was separated by dodecyl sulfate sodium-polyacrylamide gel electrophoresis (SDS-PAGE) and then transferred to polyvinylidene difluoride (PVDF) membranes. PVDF membranes were blocked with 5% (*w*/*v*) non-fat milk for 1 h at 37 °C, and then incubated with primary antibodies of caspase-3, Bcl-2 and Bax overnight at 4 °C. After being rinsed in TBS-T three times, the membranes were incubated for 2 h with horseradish peroxidase combined secondary antibody. Images were analyzed using ChemiDoc XRS with Quantity One software (Bio-Rad, Hercules, CA, USA; Figures S5–S9 of the Supplementary Materials).

### 4.10. Statistical Analysis

Data were presented as mean ± standard deviation (S.D) and analyzed with the GraphPad Prism 6.0 software. Comparisons between two groups were assessed using unpaired two-tailed Student’s *t*-test. One-way ANOVA followed Bonferroni/Dunn post-hoc test was used for multiple comparisons. *p* value <0.05 was considered statistically significant.

## 5. Conclusions

The present study illustrated that AR exerts cardioprotective effects hindering apoptosis by way of the mitochondrial signaling pathway against H/R injury. This cardioprotective effect involves in decrease the extent of mPTP opening, inhibition of H/R-injured mitochondrial cytochrome c leakage, suppression of caspase-3 activity, and enhancement of the Bcl-2/Bax ratio. AR, via ameliorating the dysfunction of mitochondria, may become a promising drug to inhibit MI/R-induced apoptosis. Its apoptosis-inhibition may have important clinical application in MI/R injury. Further experiments are executed to validate the protective efficacy of AR *in vivo* and to provide potential clues for its clinical applications.

## Supplementary Materials

Supplementary materials can be accessed at: http://www.mdpi.com/1420-3049/21/6/683/s1.
