# Supplementary material for: Clematichinenoside (AR) Attenuates Hypoxia/Reoxygenation-Induced H9c2 Cardiomyocyte Apoptosis via a Mitochondria-Mediated Signaling Pathway
Source: Molecules. 2016 May 30;21(6):683. doi: 10.3390/molecules21060683 (PMC6273438; doi:10.3390/molecules21060683)
Supplement: Supplementary file 1 [file molecules-21-00683-s001.pdf]

Supplementary Materials: Clematichinenoside (AR) Attenuates Hypoxia/Reoxygenation-Induced H9c2 Cardiomyocyte Apoptosis via a Mitochondria-Mediated Signaling Pathway

Haiyan Ding, Rong Han, Xueshan Chen, Weirong Fang, Meng Liu, Xuemei Wang, Qin Wei, Nandani Darshika Kodithuwakku and Yunman Li

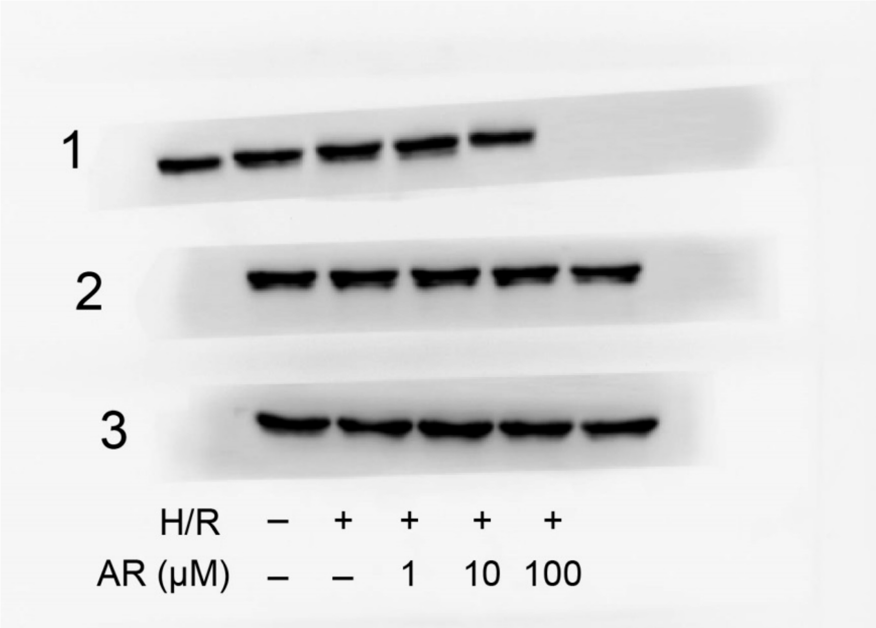

Figure S1. Western blotting detected COX IV in mitochondria.

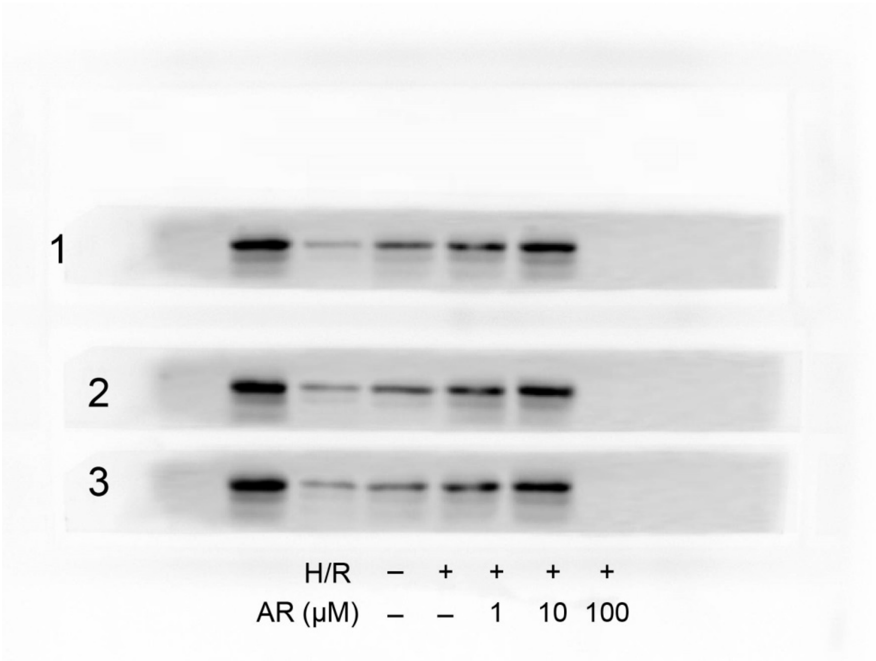

Figure S2. Western blotting detected cytochrome c in mitochondria.

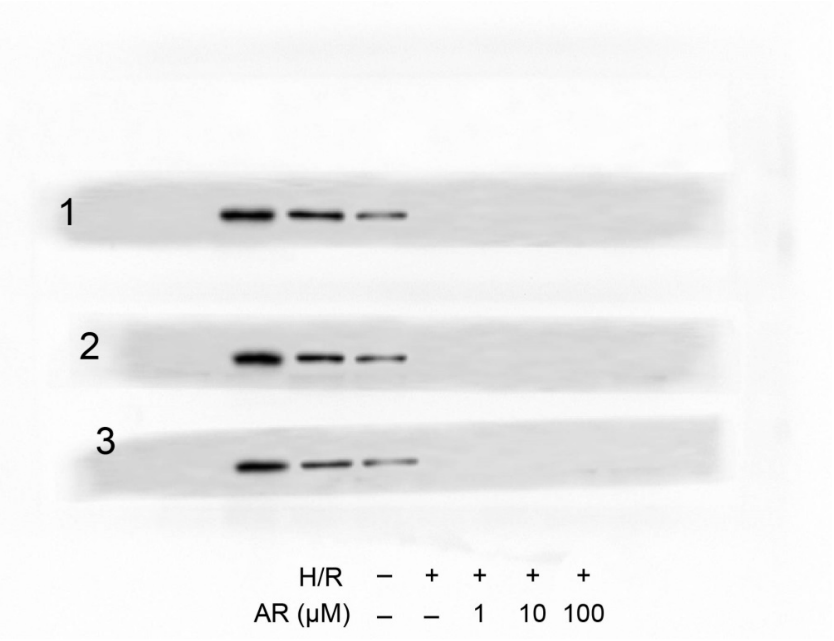

Figure S3. Cytosolic translocation of mitochondrial cytochrome c.

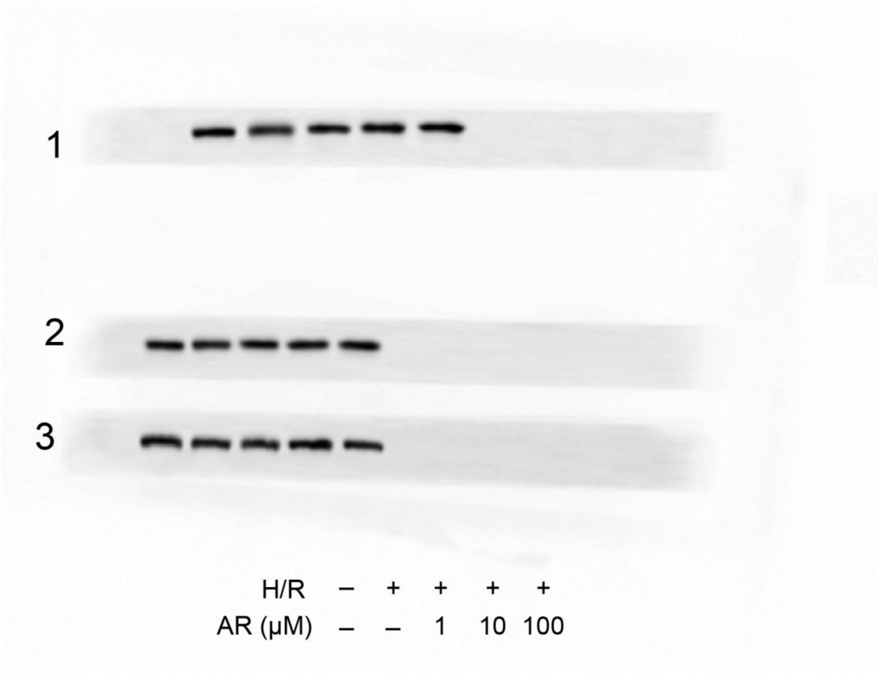

Figure S4. Western blotting detected GAPDH in cytosol.

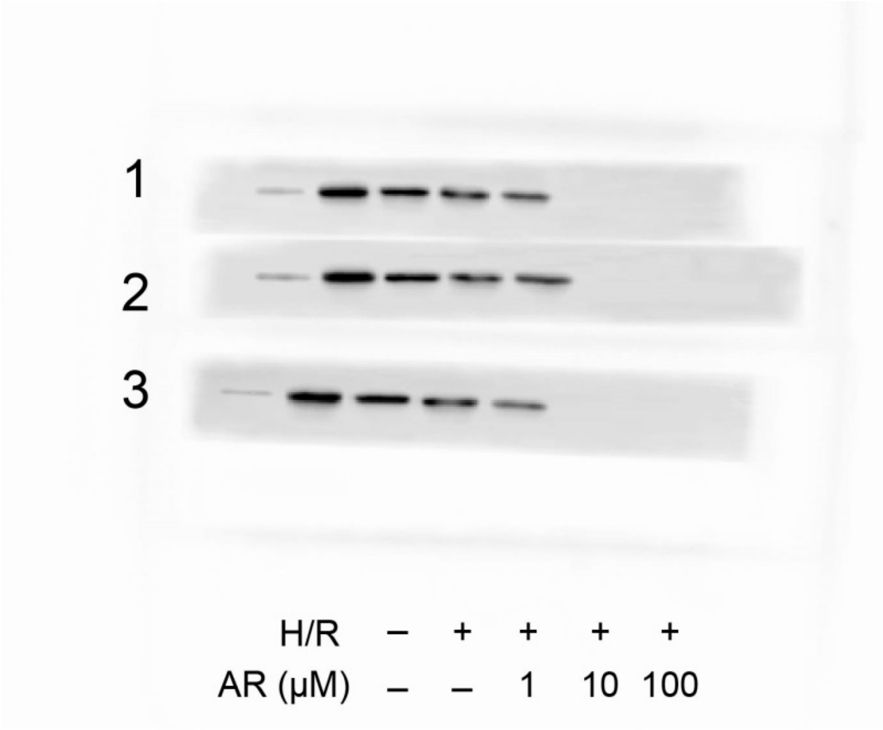

Figure S5. Western blotting detected the activity of caspase-3.

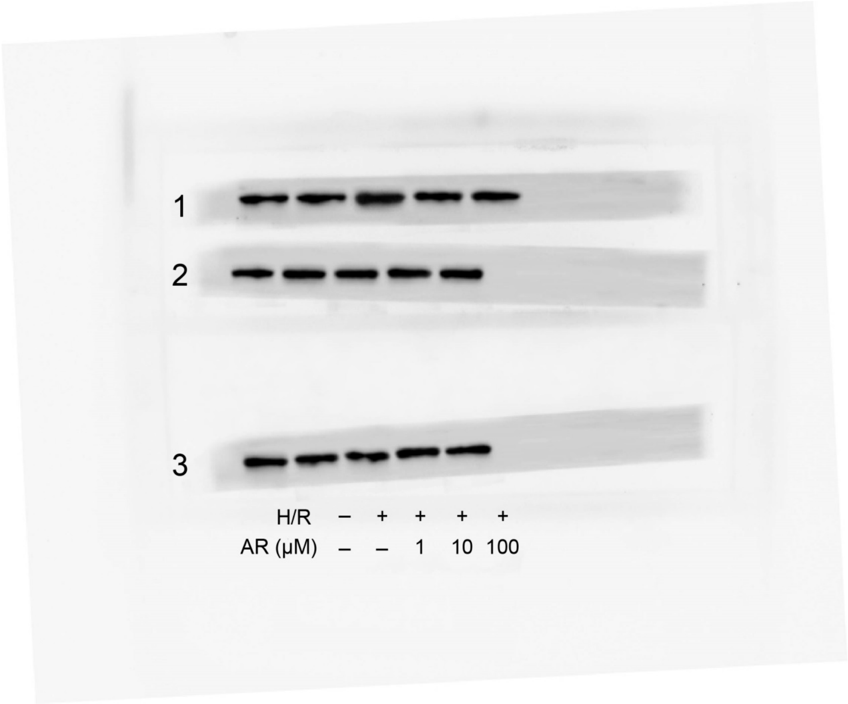

Figure S6. Western blotting detected the β-actin.

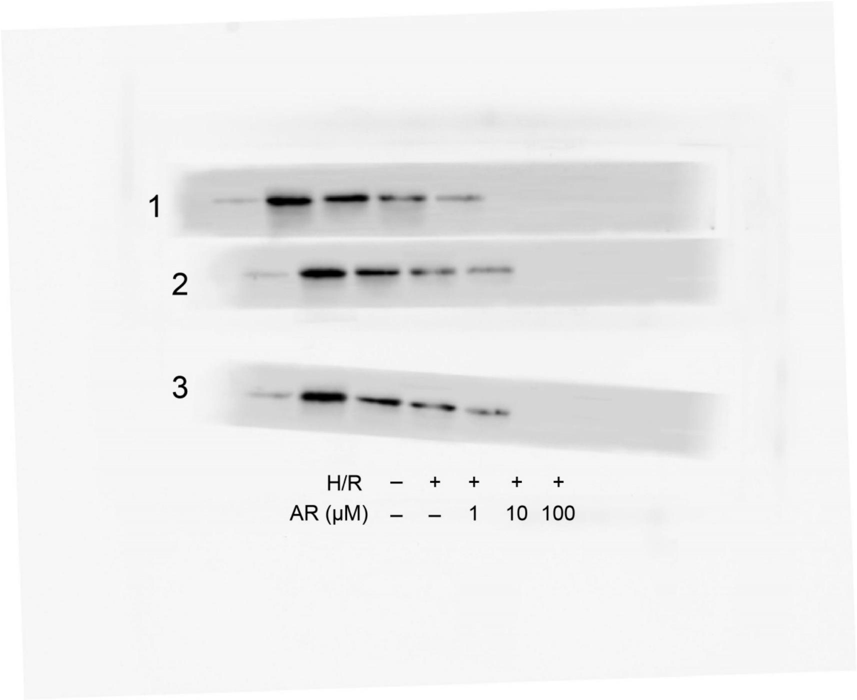

Figure S7. Western blotting detected the Bax.

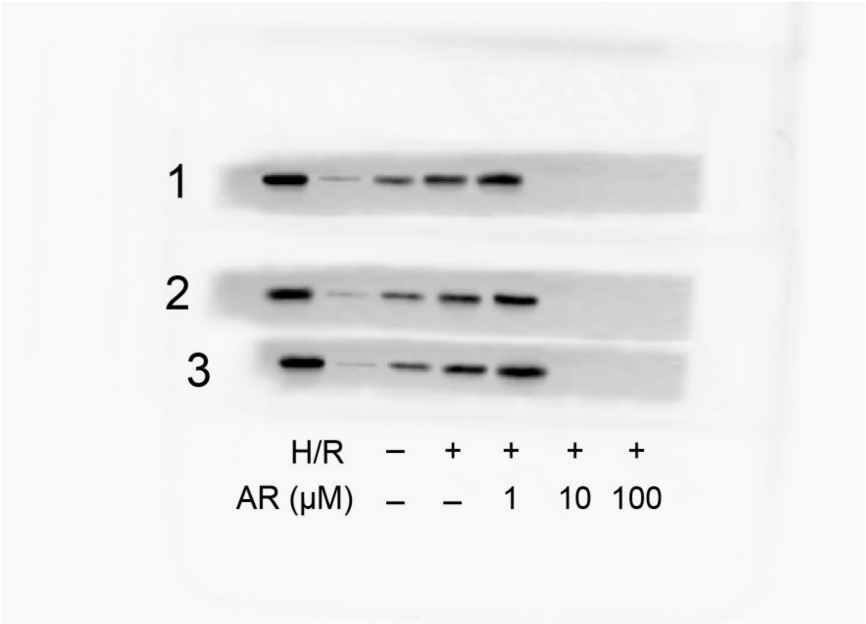

Figure S8. Western blotting detected the Bcl-2.

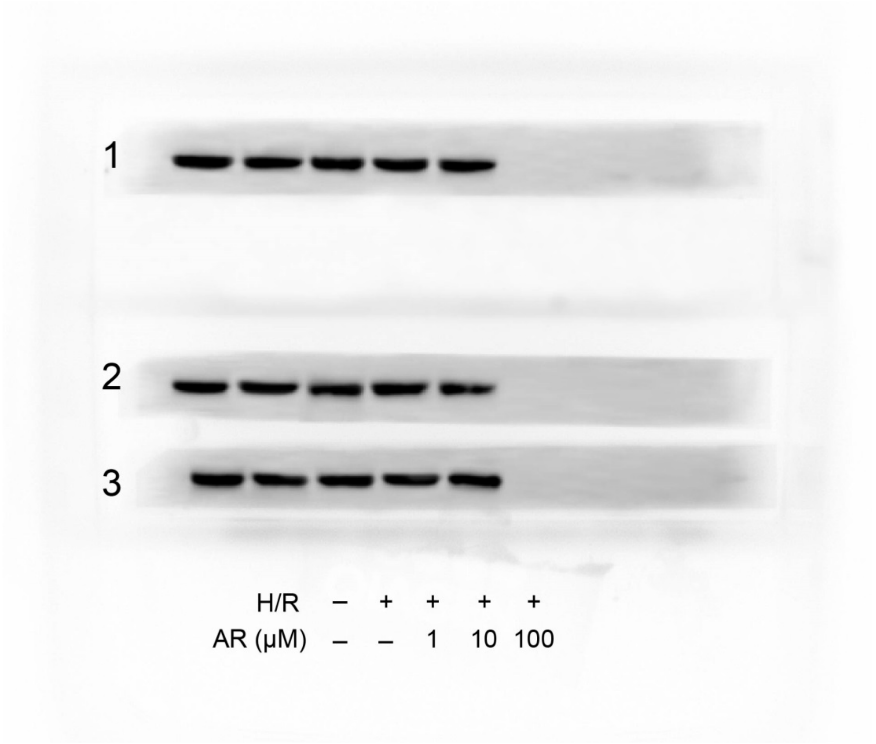

Figure S9. Western blotting detected the β-actin.
